# A proteomic clock for malignant gliomas: The role of the environment in tumorigenesis at the presymptomatic stage

**DOI:** 10.1371/journal.pone.0223558

**Published:** 2019-10-10

**Authors:** Le Zheng, Yan Zhang, Shiying Hao, Lin Chen, Zhen Sun, Chi Yan, John C. Whitin, Taichang Jang, Milton Merchant, Doff B. McElhinney, Karl G. Sylvester, Harvey J. Cohen, Lawrence Recht, Xiaoming Yao, Xuefeng B. Ling

**Affiliations:** 1 Department of Cardiothoracic Surgery, Stanford University, Stanford, California, United States of America; 2 Clinical and Translational Research Program, Betty Irene Moore Children's Heart Center, Lucile Packard Children’s Hospital, Palo Alto, California, United States of America; 3 Department of Oncology, the First Hospital of Shijiazhuang, Shijiazhuang, Hebei, China; 4 Department of Surgery, Stanford University School of Medicine, Stanford, California, United States of America; 5 Department of Pediatrics, Stanford University School of Medicine, Stanford, California, United States of America; 6 Department of Neurology and Neurological Science, Stanford University School of Medicine, Stanford, California, United States of America; University of Illinois College of Medicine, UNITED STATES

## Abstract

Malignant gliomas remain incurable with a poor prognosis despite of aggressive treatment. We have been studying the development of brain tumors in a glioma rat model, where rats develop brain tumors after prenatal exposure to ethylnitrosourea (ENU), and there is a sizable interval between when the first pathological changes are noted and tumors become detectable with MRI. Our aim to define a molecular timeline through proteomic profiling of the cerebrospinal fluid (CSF) such that brain tumor commitment can be revealed earlier than at the presymptomatic stage. A comparative proteomic approach was applied to profile CSF collected serially either before, at and after the time MRI becomes positive. Elastic net (EN) based models were developed to infer the timeline of normal or tumor development respectively, mirroring a chronology of precisely timed, “clocked”, adaptations. These CSF changes were later quantified by longitudinal entropy analyses of the EN predictive metric. False discovery rates (FDR) were computed to control the expected proportion of the EN models that are due to multiple hypothesis testing. Our ENU rat brain tumor dating EN model indicated that protein content in CSF is programmed even before tumor MRI detection. The findings of the precisely timed CSF tumor microenvironment changes at presymptomatic stages, deviation from the normal development timeline, may provide the groundwork for the understanding of adaptation of the brain environment in tumorigenesis to devise effective brain tumor management strategies.

## Introduction

Despite years of research, malignant gliomas remain incurable once detected and is the costliest cancer in terms of hospital care and lost productivity [1, 2]. We hypothesized that a better understanding of the ongoing *in situ* environmental changes preceding the development of clinical abnormalities may lead to novel diagnostic and therapeutic strategies in primary brain tumors. However, characterization of the impact of brain environmental changes in tumorigenesis has been significantly limited by the relative inaccessibility of this tissue. Although it would be difficult to visualize tumors at very early stages in brain parenchyma, cerebrospinal fluid (CSF) represents a readily accessible source that may help report brain environment adaptations before and after tumor development [3].

We have chosen to focus on the asymptomatic environment in a glioma rat preclinical model in which brain tumors invariably developed after a single *in utero* exposure to the carcinogen ethylnitrosourea (ENU) [4, 5]. This rodent model permits observational imaging and histological analysis of a tumor from its earliest, presymptomatic stages. Our findings [6, 7] suggest that early pathological changes can be detected as early 30 days of age (P30) and that tumor development occurs in a characteristic, predictable (albeit stochastic) pattern. To understand what is happening within the presymptomatic environment, we have also analyzed CSF at these early time points using a mass spectrometric proteomics profiling method. In our controlled rat model study, matched ENU- and saline-exposed rats’ CSF proteomics changes were quantified at approximately 30, 60, 90, 120, 150 days of age (P30, P60, P90, P120, P150). The profiling of the samples are described in Table 1. We previously identified the presence of increased albumin, fragments of CSF proteins, and glutathione-related posttranslational modifications of transthyretin protein in temporal association with the development of cellular hyperplasia [8, 9]. In addition, we applied our transition-based network entropy (TNE) method and identified a dynamic driver network (DDN) of CSF proteins related with the emerging tumorigenesis progressing from the non-hyperplasia state, and the critical transition state prior to impending hyperplasia [8]. Our analysis indicates that there are major presymptomatic environmental changes.

**Table 1:** Cohorts of case and control rats.

Times	Sample description		
	Case (samples)	Control (samples)	Features
**Day 30**	13	11	247
**Day 60**	16	16	
**Day 90**	22	23	
**Day 120**	6	5	
**Day 150**	7	5	

**Fig 1:**
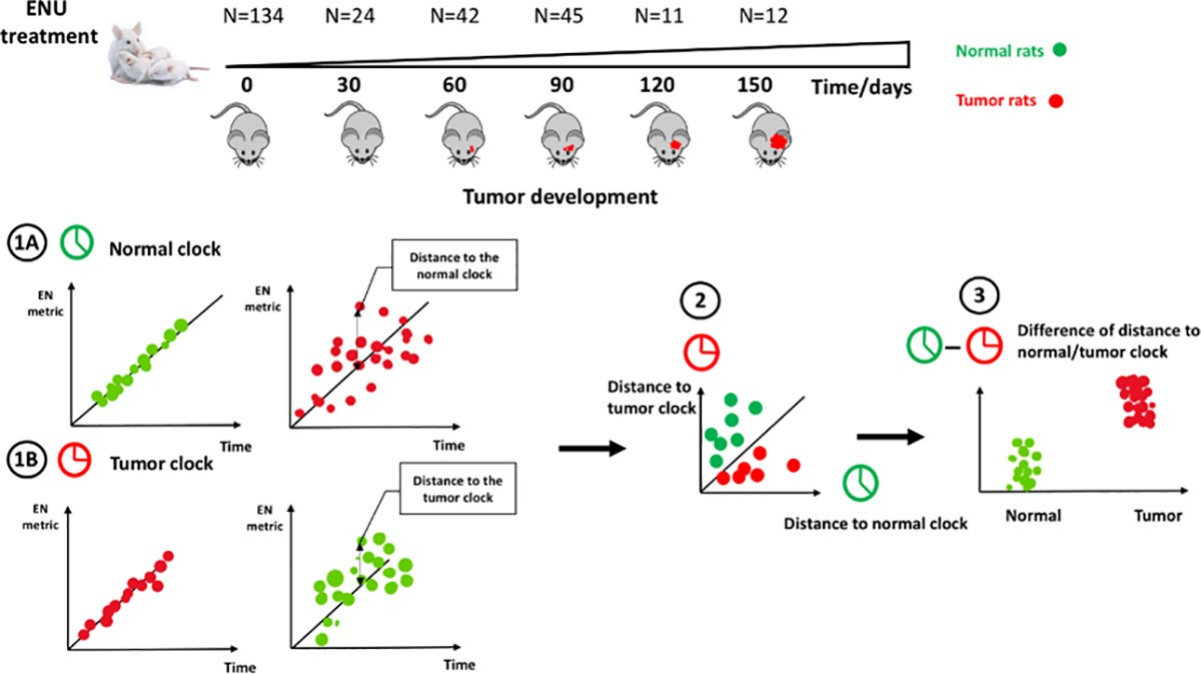
Study outline of the development of the “clock”. (1A) Development of the normal clock. (1B) Development of the tumor clock. (2) & (3) The distance to the normal clock and the tumor clock can be used as a criterion to predict the tumor rats before the symptom appears.

The major aim of this study was to examine whether analysis of cerebrospinal fluid (CSF) collected before and after MRI detectable brain tumor development would allow the identification of a protein panel capable of tightly tracking the timed events of tumor initiation, promotion, and progression. We hypothesize that the precisely timed changes in the CSF proteome mirrors a proteomic clock for malignant gliomas (Fig 1), and that deviation from the normal rat development chronology, provides the groundwork for the understanding of the seed and soil relationship in brain primary tumorigenesis. This information may help device effective brain tumor early detection and therapeutic strategies.

## Materials and methods

### Study design

This study was designed, and shown in Fig 1, to determine whether a timed CSF proteome change can be detectable during tumor growth in a rat model.

### ENU exposed anaimla model

ENU produces N- and O-ethylation damage to DNA to which targeted cells then apply repair mechanisms. This insult occurs rapidl since drug is cleared within minutes [4, 5]. However, repair processes soon ensue over a longer period that result in a random mutagenesis including deletions, substitutions and translocations [10, 11]. Rat brains removed acutely after exposure in the late embryonic period (usually between E16 and E19) reveal a marked increase in apoptotic rate for 48–72 hours primarily in the subventricular zone (SVZ), after which brains then become indistinguishable from normal controls at birth. Then ensues a prolonged asymptomatic interval that can last many months depending on dosage [1], after which rats invariably die of brain tumors. ENU-induced glioma. ENU administration, rat CSF collection and subsequent histological analysis were as previously described [8]. CSF proteomic profiling and subsequent data analysis were as previously performed [9, 12–13]. Case (ENU) and control rat handling was in accordance with guidelines for animal safety and welfare. Rat CSF proteomic experiments were approved by the Administration Panel on Laboratory Animal Care, the accredited body at Stanford.

### Bayesian inference and early brain tumor detection

Bayesian inference [14] is one of the two broad categories of interpretations in statistical inference. Specifically, for a given observation (or rat) *x*, the probability of the observation belongs to the normal group is *P*_*r*_(*normal*|*x*), whereas the probability of the observation belongs to the normal group is *P*_*r*_(*tumor*|*x*). Let

$$\Delta P_{r}=P_{r}(tumor|x)-P_{r}\left(normal\right|x),$$ (1)

It is easy to see that the more Δ*P*_*r*_ approaches -1.0, the observation can be classified in the normal group with more confidence. Similarly, the more Δ*P*_*r*_ approaches +1.0, the observation can be classified in the tumor group with more confidence. However, it is difficult to compute *P*_*r*_(*normal*|*x*) or *P*_*r*_(*tumor*|*x*) directly from the data. Follow the Bayesian theorem, we can get

$$P_{r}(normal|x)=\frac{P_{r}(normal)}{P_{r}(x)}\times P_{r}(x|normal),$$ (2)

$$P_{r}(tumor|x)=\frac{P_{r}(tumor)}{P_{r}(x)}\times P_{r}(x|tumor),$$ (3)

In (2) and (3), *P*_*r*_(*normal*) and *P*_*r*_(*tumor*) are the probability of normal and tumor, respectively. In our study,

$$P_{r}(normal)=\#control/(\#control+\#case)\approx 0.48,$$ (4)

$$P_{r}(tumor)=\#case/(\#control+\#case)\approx 0.52,$$ (5)

Omitting the difference of *P*_*r*_(*normal*) and *P*_*r*_(*tumor*), we have

$$\Delta P_{r}=P_{r}(tumor|x)-P_{r}\left(normal\right|x)∼P_{r}(x|tumor)-P_{r}(x|normal),$$ (6)

Note that *P*_*r*_(*x*|*tumor*) is the probability of the observation given the tumor group, which can be interpreted as the randomness criteria in the tumor clock (Fig 1.1B). Similarly, *P*_*r*_(*x*|*normal*) is the probability of the observation given the normal group, which can be interpreted as the randomness criteria in the normal clock (Fig 1.1A). As stated above, the closer an observation is to the regression line in the tumor clock, the more it is subject to the tumor clock, which is less random in the tumor clock, and vice versa. Therefore, (6) can be derived as (Fig 1.2 and 1.3)

$$\Delta P_{r}∼P_{r}(x|tumor)-P_{r}(x|normal)∼dis(xtonormal)-dis(xtotumor),$$ (7)

Therefore, early brain tumor can be detected using the criteria in (7) derived from the Bayesian Inference theorem.

### Feature selection and statistical analysis

To assess the relative contributions of each of the m/Z features to the normal/tumor clock, the Elastic Net (EN) algorithm [15] is used as a feature selection and regression tool, since the EN algorithm can automatically balances regression algorithm against the number of markers in the normal/tumor clock estimation. For a matrix *X* of all protein intensities of peaks and a vector of estimated age at time of sampling *Y*, a multivariate model was developed for each normal rat to minimize the loss function from the EN algorithm:

$$L(\beta )={\left|Y-X\beta \right|}^{2}+\gamma_{1}{\left|\beta \right|}_{1}+\gamma_{2}{\left|\beta \right|}_{2},$$ (8)

In (8), the first term is the least square error for the regression, which represents the overall differences between the actual and the predicted clock of the controls. The second term performs the *L*_1_ regularization to limit the number of markers are used. The third term performs the *L*_2_ regularization to allow the inclusion of highly correlated and potentially biologically relevant features. The parameters *γ*_1_ and *γ*_2_ were optimized using cross-validation.

### Global false discovery

The R-square difference of the clock in the normal group and the tumor group indicated that the tumor group did not follow the normal clock. In order to prove that this difference did not come from statistical randomness, we estimated the False Discovery Rate (FDR) in concurrent statistical tests, of the same size as our normal and tumor group; in multiple permutated “random” training data sets were constructed [13].

## Results

### Sample characteristics

Nestin^+^ cell clusters and microtumors were assessed in 64 ENU-exposed rats on 30, 60, 90, 120, and 150 days of age (Fig 1). CSF was obtained as previously [9] described from the cisterna magna from 124 exposed and control rats at 30, 60, 90, 120, and 150 days and then analyzed by mass spectrometry to profile CSF proteomes. Differently expressed protein peaks were isolated and identified. All the analyses are performed with R machine learning tool available at https://cran.r-project.org/.

### The CSF proteome normal clock and tumor clock

**Fig 2:**
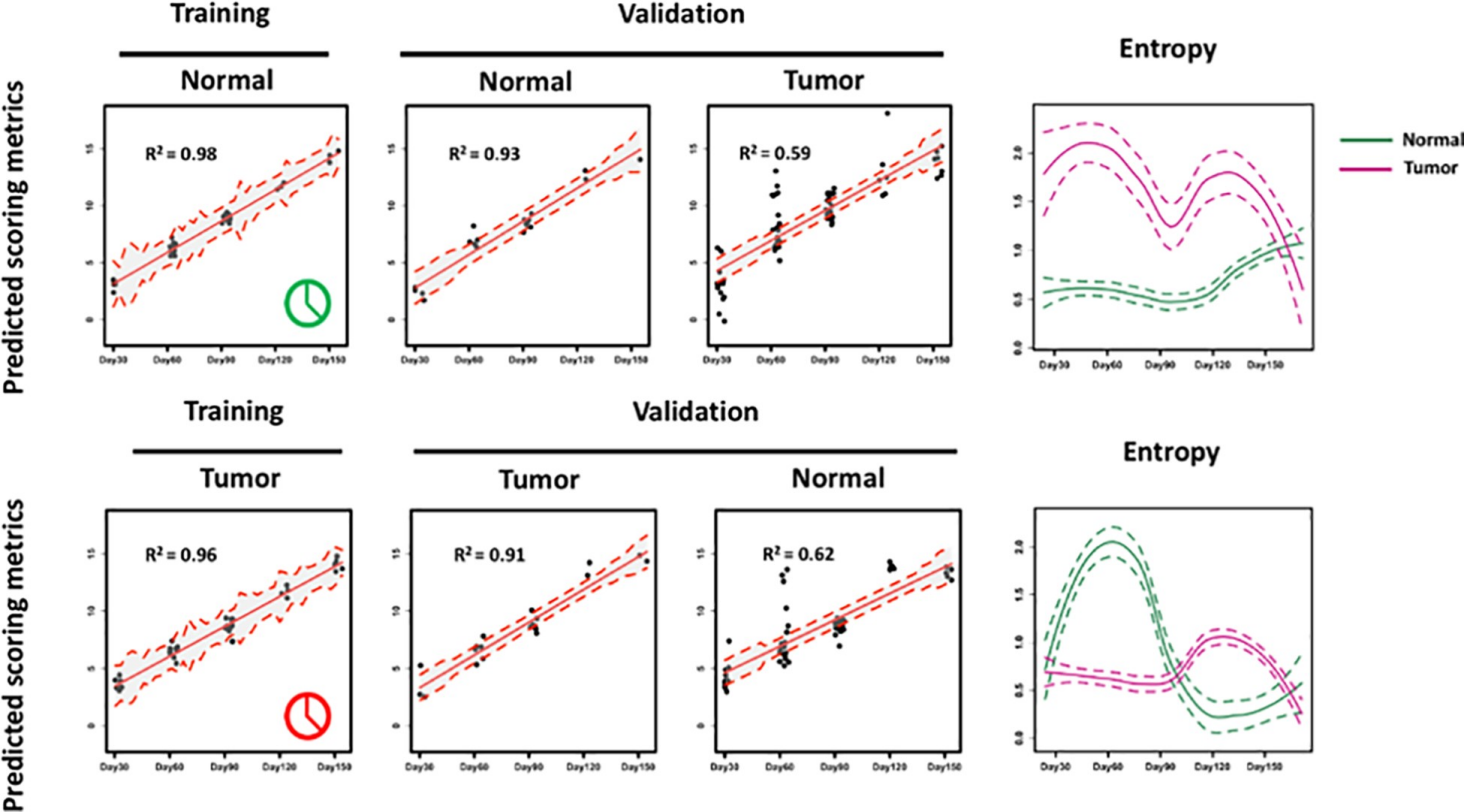
The normal clock and the tumor clock development with real data. With EN algorithm, we modeled a precisely timed CSF protein expression programmed dynamically over the growth of the normal rats, mirroring a normal clock of aging. Also with EN algorithm, we identified a precisely timed CSF proteome pattern programmed over the tumor development of these ENU exposed rats, mirroring a tumor clock.

We randomly split the control rats into 2/3 (training cohort) and 1/3 (testing cohort). Utilizing the EN algorithm, we first developed a predictive model with the training cohort of the control rats that was strongly associated with age (R^2^ = 0.98, Fig 2 upper left panel). The validity of the EN model was tested in the testing cohort of the control rats (R^2^ = 0.93, Fig 2 upper middle panel). Together, the analysis identified a timed CSF protein expression programmed dynamically over the age of normal rats.

To develop a tumor clock, we also randomly split the ENU exposed rats into 2/3 training cohort and 1/3 testing cohort. With EN algorithm, as for the control rats we first developed a predictive model with a training cohort of the exposed rats such that the EN metric is linearly correlated with the CSF sampling time (R^2^ = 0.96, Fig 2 lower left panel). The validity of the EN model was independently tested in the testing cohort of the exposed rats (R^2^ = 0.91, Fig 2 lower middle panel). Together, the analysis identified a timed CSF proteome pattern programmed over tumor development of these exposed rats, mirroring a tumor clock.

### The normal/tumor clock malfunctions in exposed/control rats

Given that the normal clock dates the normal development, we hypothesized that EN predictive model can contextualize abnormal phenotypes due to a malfunctioned brain microenvironment such as that seen in the exposed rats. In contrast, the normal clock (training R^2^ = 0.98 and testing R^2^ = 0.93, accurately predicting the growth of the normal rats) displayed a significant drop of the R^2^ when tested on the exposed rats (R^2^ = 0.59, Fig 2 upper middle panel). Similarly, the tumor clock (training R^2^ = 0.96 and testing R^2^ = 0.91 accurately predicting the growth of the exposed rats) displayed a similar decrease of R^2^ when tested on the control rats (R^2^ = 0.62, Fig 2 lower middle panel). We conclude that the normal or tumor clock can function properly if applied to the targeted group of normal or tumor rats. If cross applied, predictive power (measured by R^2^) of the clocks was diminished.

### False discovery analysis of the “clocks”

**Fig 3:**
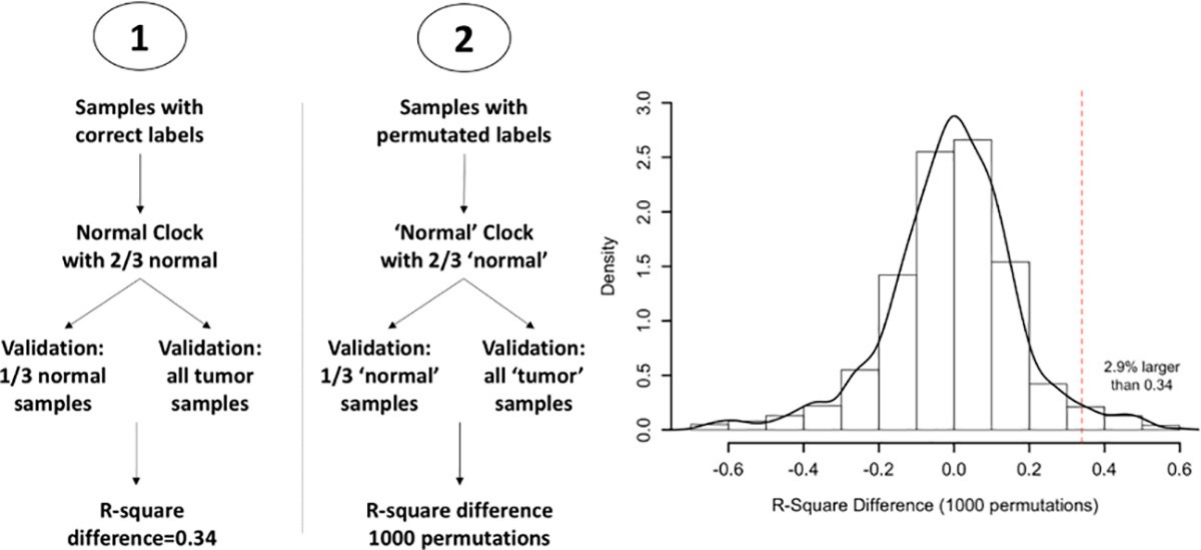
The False discovery analysis of the “clock”. The class labels of our training rat samples were permutated 1000 times such that each time every sample would be randomly assigned a new class label. FDR was calculated as the number of the R-square differences greater than that of real labelled samples by the permutation time.

The class labels of our training rat samples were permutated 1000 times such that each time every sample would be randomly assigned a new class label. For each of the 1000 simulated sets, 2/3 of the labelled 'normal' samples were used to construct the clock model, and the R-square differences between the rest 1/3 'normal' samples and all the 'tumor' samples were computed and recorded (Fig 3 left panel). The density distribution was plotted in Fig 3 right panel. FDR was calculated as the number of the R-square differences greater than that of real labelled samples by the permutation time. As shown in Fig 3 right panel, the FDR was estimated as 2.9%, which supported the notion that the tumor samples did not follow the normal clock is unlikely to be the outcome of chance.

### Characterization of the entropy kinetics

Given that our normal/tumor clocks date the timed CSF proteome changes in normal/ENU-exposed rats, we hypothesizethe entropy, measuring the longitudinal chaotic dynamics, i.e. deviation kinetics, of the EN predictive metric, should be disparate between the normal and exposed dysfunctional “clocks”. Shown in the right panel of Fig 2, we compared the timed entropy pattern changes of the EN predictive metric between the normal and ENU-exposed rats respectively. In early stages (30 and 60 days of age), the entropy peaks at day 60 after birth, which is consistent with our previous critical transition paper [1]. Another observation is that in the normal clock, the entropy of the ENU-exposed rats is much larger than the normal controls, and vice versa. At 90 days of age, the entropy reaches a local minimum. MRI detectable tumors appear afterwards, and the degree of chaos decreases again indicating the decrease of both entropies in Fig 2.

### Two dimensional predictor of the tumorigenesis outcome

**Fig 4:**
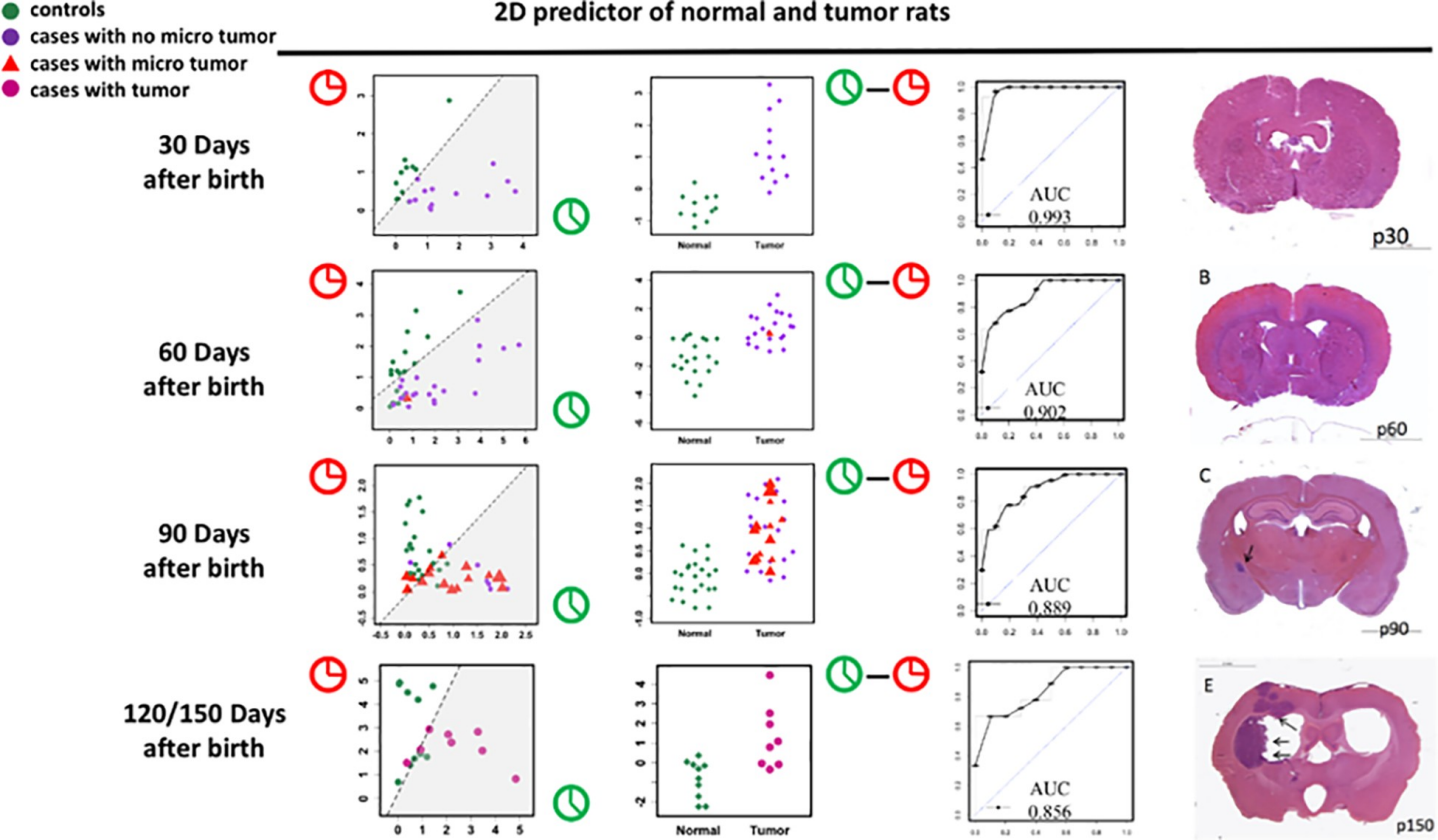
The performance of the 2D predictor. The 2D predictor to predict tumor and normal rats using data collected from 30 days, 60 days, 90 days, and 120/150 days after birth, respectively. Each green circle represents a rat from the control group, each purple circle denotes a rat with no microtumor from the ENU exposed group while each red triangle indicates the rat from the ENU exposed group that has been observed with microtumors, and the violet circles are the rats that already have tumors in the brain.

**Fig 5:**
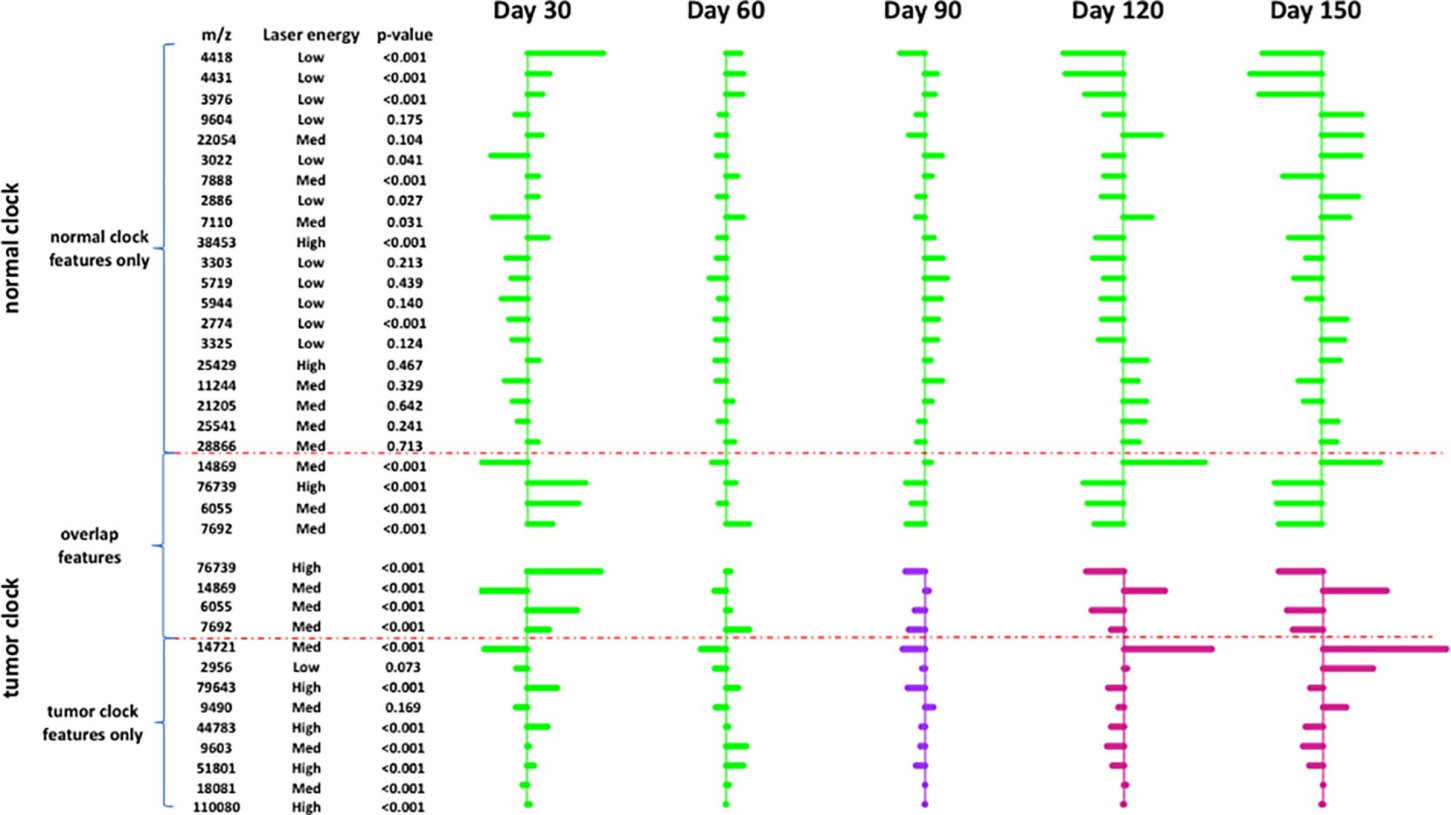
Proteomic changes in rat CSF at different time points between tumor and normal clocks. The figure shows the coefficients of the selected analyte peaks in the normal clock and in the tumor clock, which reflects the proteomic changes across different timestamps. The p-value is used to measure the proteomic changes in day 30 and day 150.

With the normal and tumor clock, we developed a two dimensional (2D) predictor to predict the tumor outcomes of the exposed rats even before any MRI detected microtumors. To quantify the deviation of tested subjects from the clock model, sampled testing subjects’ distances to either the normal clock or the tumor clock were analyzed on a 2D plot at 30, 60, 90, 120/150 days (Fig 4, after 120 days all the microtumor developed to obvious tumors). Shown in Fig 4 right panel, at 30 days of age, there is no MRI detected mirotumors in either the control or ENU-exposed rats. However, entropy dynamics analyses with the normal and tumor clock revealed clear differences from the first longitudinal sampling point (day 30), therefore, we hypothesized that integrative analytics of both normal and tumor metrics can lead to a 2D classifier to predict future MRI detectable brain tumor (AUC 0.993, Fig 4 line 1). Our MRI analysis revealed, at 60 or 90 days of age, microtumors in the ENU-exposed rats. The 2D predictor works well with an AUC of 0.902 and 0.889 (Fig 4 line 2 and 3). At 120 or 150 days of age, all the rats from the ENU-exposed group have tumors but the performance of the 2D predictor starts to deteriorate and get worse with time. The coeffcients of the selected analyte peaks at different timestamps is shown in Fig 5. Since the experimental method is untargeted, it is hard to identify the analyte peaks. This is a limitation of the study.

## Discussion

To provide preclinical data to help the exploration of the mechanisms underlying gliomas pathophysiology, we have been studying the development of brain tumor in an animal model where rats develop brain tumors after prenatal exposure to ENU at around six months of age. Our proteomic analysis revealed timed CSF proteome changes in the normal and ENU exposed rat cohorts respectively. Elastic net models were developed mirroring “proteomic clocks” that strongly track either the normal development or tumor initiation, promotion and progression. Integrative analysis of the deviations from either the normal or glioma development chronologies led to a robust 2D classifier predictive of tumor development prior to the development of measurable tumors.

Despite the fact our findings revealed the disruption of the normal chronology at a stage where there is no obvious lesion and pathological differentiation in ENU exposed rats, we cannot delineate the causative relationship between the initiating tumor cells (seed) harboring tumorigenic mutations and the programmed brain microenvironment (soil) adaptations due to systemic response to injury or tumor. By design, the first sampling time point is day 30, therefore, there are limited sampling points to characterize the chronological proteomic changes before the MRI detectable pathological lesions. In addition, it is unlikely that all disruptive proteomic patterns prognostic and/or indicative of tumorigenesis and tumor development will become manifest as deviations from the normal chronological profile.

**Fig 6:**
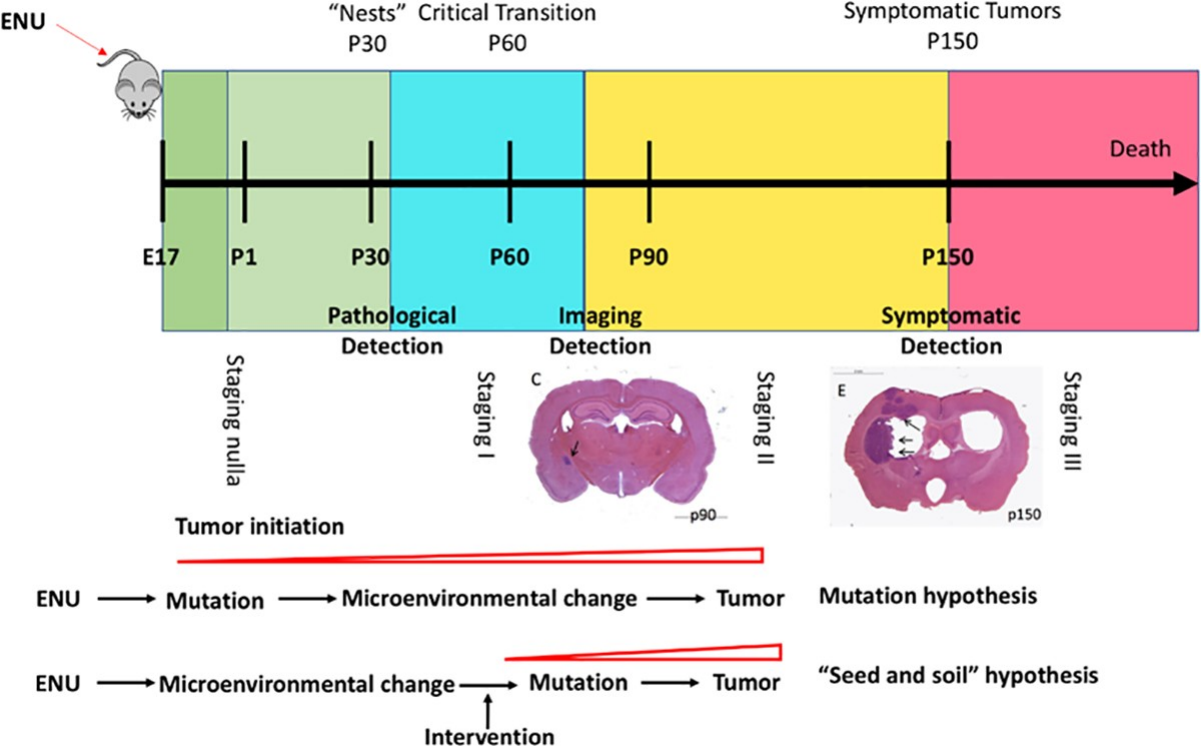
A four-stage timeline of brain tumor development in ENU rats model. Stage nulla represents the interval from the tumorigenesis of the first initiating tumor cell harboring tumorigenic mutation until the first pathological changes are noted; Stage I represents the interval from when the first pathological changes are noted until pathological lesions are detectable by MR, Stage II represents the interval between initial detection on MRI and clinical symptomatology and Stage III represents the stage when symptoms appear (which is the usual stage at which these tumors are first detected clinically).

Nevertheless, our proteomic analysis revealed a much earlier timeline for the development of brain tumors before the late stage abnormalities, hyperplastic lesions detectable by MR imaging usually starting between 90 and 120 days of age. The elastic-net based tumor clocks quantitatively track the CSF proteome changes and the deviations from the normal chronology, destined to harbor tumors as early as 30 days of age, several weeks before even cellular hyperplasia becomes evident. The entropy dynamics analyses with the normal and tumor clock revealed differences from the first longitudinal sampling point (day 30), and this finding was validated by a 2D classifier of day 30 to predict future MRI detectable brain tumor (AUC 0.993). Therefore, the entropic analyses quantified the chronological differences contrasting the tumor and normal control cohorts before tumors become detectable with MRI. Based on our longitudinal clock findings of tumor microenvironment differentiation before the clinical lesions, we propose a four stage timeline of brain tumor development (Fig 6): Stage nulla represents the interval from the tumorigenesis of the first initiating tumor cell harboring tumorigenic mutation until the first pathological changes are noted; Stage I represents the interval from when the first pathological changes are noted until pathological lesions are detectable by MR, Stage II represents the interval between initial detection on MRI and clinical symptomatology and Stage III represents the stage when symptoms appear (which is the usual stage at which these tumors are first detected clinically). Our quantitative clock analyses thus exposed a potential intervention window, Stage nulla, when there are no pathological changes noted, however, microenvironment might have adapated preparing the emerging tumoral lesions.

Extrapolation from the rat, in which there is a long interval between initial appearance of pathological changes and lesion detectability on MRI (approximately 40 days, the equivalent of almost four years in the human life span and very consistent with the observed time to onset of brain tumors in the childhood cancer survivor [16]. With our definition of the Stage nulla in gliomas, we propose that the best chance for cure of malignant brain tumor may lie in an early detection and treatment strategy.

Given that early detection of malignant glioma is still an unmet clinical problem, analysis of the CSF proteomes along the tumor development could help not only understand the molecular mechanism underlying tumorigenesis and pathogenesis, but also lead to robust protein panels to allow early diagnosis at pre-symptom stage. In order to translate the findings of current animal model results to clinical application, validation study by targeted protein analysis of the tumor clock features in human glioma subject CSFs is essential.

For such an approach to work, one must define and translate our preclinical findings of this window to clinical setting for therapeutic opportunity. In such scenario, if early detection of the microenvironment changes could be detected using our tumor clock methodologies, prediction and prevention of tumor metastasis could be achieved. Given that the ENU-model is also ideal for comparing early and late administration of a particular treatment on outcome, we can launch different assessment strategies by examining the overall effect on outcomes. In that regard, our findings shall provide the groundwork for the understanding of the tumorigenesis at the asymptomatic stage to device effective brain tumor management strategies.

Despite years of research, malignant gliomas remain incurable once detected and is the costliest cancer in terms of hospital care and lost productivity. The possibility of asymptomatic brain tumors has received little discussion in the literature and there is an absence of evidence sustaining the clinical utility of brain tumor identification prior to magnetic resonance imaging (MRI) detectable lesion, in terms of clinical and economic advantages [17]. To date, studies have almost exclusively examined samples drawn from patients in whom the brain tumor is already clinically evident, which makes it difficult to distinguish what is a result of the brain tumor itself versus other effects including brain microenvironment disruption. Our long term goal is to define a molecular timeline through the multi-proteomics profiling of the brain microenvironment such that brain tumor commitment can be revealed earlier at an asymptomatic stage. Our future study would be more focused upon the diverse proteome especially in a complex dynamic environment encountered in glioma as shown in recent literature [18].

## Supporting information

S1 Table The data used in the anaylsis is shown in S1 Table. (XLSX)
